# Tripartite Motif-Containing 2, a Glutamine Metabolism-Associated Protein, Predicts Poor Patient Outcome in Triple-Negative Breast Cancer Treated with Chemotherapy

**DOI:** 10.3390/cancers16111949

**Published:** 2024-05-21

**Authors:** Brendah K. Masisi, Rokaya El Ansari, Lutfi Alfarsi, Ali Fakroun, Busra Erkan, Asmaa Ibrahim, Michael Toss, Ian O. Ellis, Emad A. Rakha, Andrew R. Green

**Affiliations:** 1 Nottingham Breast Cancer Research Centre, Academic Unit for Translational Medical Sciences, School of Medicine, University of Nottingham Biodiscovery Institute, University Park, Nottingham NG7 2RD, UK; 2 Cellular Pathology, Nottingham University Hospitals NHS Trust, Nottingham City Hospital, Hucknall Road, Nottingham NG5 1PB, UK

**Keywords:** glutaminase (GLS), tripartite motif-containing 2 (TRIM2), triple-negative breast cancer (TNBC), prognostic significance, patient outcome, molecular mechanism

## Abstract

**Simple Summary:**

Triple-negative breast cancer (TNBC) is an aggressive form of breast cancer with limited treatment options. This study looked for markers in breast cancer cells linked to glutamine metabolism, an important fuel source for cancer cells. It found a protein called TRIM2 to be highly expressed alongside a key glutamine metabolism enzyme in TNBC. High levels of TRIM2 were linked to a worse chance of spread of cancer to distant sites. This association was particularly strong in patients who received chemotherapy. These findings suggest TRIM2 could be a valuable prognostic marker for TNBC patients, especially those undergoing chemotherapy. Further research is needed to understand how TRIM2 functions and its connection to glutamine metabolism in breast cancer.

**Abstract:**

Background: Breast cancer (BC) remains heterogeneous in terms of prognosis and response to treatment. Metabolic reprogramming is a critical part of oncogenesis and a potential therapeutic target. Glutaminase (GLS), which generates glutamate from glutamine, plays a role in triple-negative breast cancer (TNBC). However, targeting GLS directly may be difficult, as it is essential for normal cell function. This study aimed to determine potential targets in BC associated with glutamine metabolism and evaluate their prognostic value in BC. Methods: The iNET model was used to identify genes in BC that are associated with *GLS* using RNA-sequencing data. The prognostic significance of tripartite motif-containing 2 (*TRIM2*) mRNA was assessed in BC transcriptomic data (*n* = 16,575), and TRIM2 protein expression was evaluated using immunohistochemistry (*n* = 749) in patients with early-stage invasive breast cancer with long-term follow-up. The associations between TRIM2 expression and clinicopathological features and patient outcomes were evaluated. Results: Pathway analysis identified *TRIM2* expression as an important gene co-expressed with high *GLS* expression in BC. High *TRIM2* mRNA and TRIM2 protein expression were associated with TNBC (*p* < 0.01). TRIM2 was a predictor of poor distant metastasis-free survival (DMFS) in TNBC (*p* < 0.01), and this was independent of established prognostic factors (*p* < 0.05), particularly in those who received chemotherapy (*p* < 0.05). In addition, TRIM2 was a predictor of shorter DMFS in TNBC treated with chemotherapy (*p* < 0.01). Conclusions: This study provides evidence of an association between TRIM2 and poor patient outcomes in TNBC, especially those treated with chemotherapy. The molecular mechanisms and functional behaviour of TRIM2 and the functional link with GLS in BC warrant further exploration using in vitro models.

## 1. Introduction

Breast cancer (BC) exhibits significant heterogeneity with different molecular subtypes, with the main luminal subtype accounting for approximately 75% [1]. Although numerous genes and proteins act as prognostic and predictive factors, only a few remain decisive for treatment, which is reflected in current stratification [2,3]. 

Estrogen receptor negative (ER-)/progesterone receptor negative (PR-)/human epidermal receptor growth factor 2 negative (HER2-), or triple-negative BC (TNBC) account for approximately 15% of BC. TNBCs are typically of high histological grade and are the most aggressive BC subtype with the worst prognosis. The current treatment of TNBC remains a clinical and scientific challenge.

Metabolic reprogramming is one of the key characteristics in cancer cell proliferation and tumour growth [4] that has attracted lots of attention as a promising field of therapy. Reprogrammed cellular metabolism in BC involves increased glucose intake and glutamine addiction. Glutamine consumption rate increases in cancer cells to provide intermediates required for biosynthetic pathways [5,6]. This reprogramming is due to changes in regulatory proteins such as oncogenes and/or oncogenic signalling pathways and control tumour suppressors, resulting in metabolic pathway changes. 

We recently confirmed that glutaminase (GLS), which generates glutamate from glutamine, plays a role in TNBC biology [7]. However, targeting GLS directly may be difficult, as it is essential for normal cell function. In addition, it is highly regulated, being controlled by a variety of factors including nutrient availability, growth factors, and cellular stress [8]. There are also multiple isoforms that potentially have different roles [7]. Therefore, identifying and targeting signalling pathways of GLS is an important strategy for discovering new biomarkers and potential molecular targets for TNBC treatment. 

This study identifies tripartite motif-containing 2 (*TRIM2*) as being highly related to GLS signalling in BC. The tripartite motif (TRIM) family of proteins contains a conserved “RBCC” motif, which includes the RING domain, the B-box motif, and the coiled-coil region. TRIM2 belongs to the TRIM family of proteins, which has more than 80 members. *TRIM2* is an 81 kDa multi-domain protein, and the gene is located at 4q31.3 [9,10]. TRIM proteins, one of the subfamilies of the RING-type E3 ubiquitin ligases, are involved in a broad range of biological processes, and their alterations are associated with disease incidence and progression relevant to the development of common cancer [9,11]. Several members of the TRIM family have previously been implicated as either tumour suppressors or oncogenes in BC, including TRIM16, TRIM24, TRIM32, TRIM33, TRIM45, TRIM47, and TRIM59 [12]. There is, however, scant information on the role of TRIM2 in BC. In ER-positive BC, TRIM2 has been linked with tamoxifen resistance by mediating apoptosis [13]. In ER-negative, basal-like BC, TRIM2 expression is associated with the TNBC-associated nuclear transcription factor SOX10 [14].

Building on the discovery that *TRIM2* gene expression is correlated with GLS signalling, this study further analyses the potential significance of the *TRIM2* gene and TRIM2 protein expression in BC progression, with a specific focus on TNBC, using a comprehensive collection of primary BC samples.

## 2. Materials and Methods

### 2.1. Biological Co-Expression Networks 

The iNET model version 2.0 (https://inetmodels.com accessed on 14 February 2023) was used to identify genes in BC that directly interacted with *GLS* using RNA sequencing data [15]. This software was used to create an interactive visualisation and database of multi-omics data based on the Z-score and significant *p* value of each target gene against *GLS*. Genes were ranked according to the highest score and a statistically significant *p* value. 

### 2.2. Study Cohorts

#### Breast Cancer Gene-Expression Miner

*TRIM2* gene expression was evaluated using Breast Cancer Gene-Expression Miner version 5.0 online DNA microarray (*n* = 11,552) and RNA sequencing datasets (*n* = 5023) (http://bcgenex.centregauducheau.fr/ accessed on 3 December 2023). This statistical mining tool, which includes the METABRIC and TCGA studies, offers the possibility to evaluate the correlation, differential expression, and prognostic significance of genes in BC. The dichotomisation of gene expression for prognostic analysis utilised the ‘optimal’ criterion.

### 2.3. Nottingham BC Series

TRIM2 protein expression was assessed in primary invasive breast tumours from a consecutive series of patients with long-term follow-up (*n* = 749). This is a well-characterised cohort of early-stage primary operable invasive BC cases treated at Nottingham University Hospital, NHS Trust, UK, between 1989 and 2006, as previously described [12]. All samples from Nottingham used in this study were pseudo-anonymised and stored in compliance with the UK Human Tissue Act. Protein expression of TRIM2 was assessed using immunohistochemistry (IHC) and tissue microarrays (TMAs) prepared from the Nottingham BC cohort. The clinicopathological profiles of the study samples including tumour size, tumour grade, nodal stage, vascular invasion (VI) status, and molecular subtypes were available. The hormonal receptor expression profiles and outcome data were also recorded. The cohort characteristics are summarised in Supplementary Table S1. Protein data for ER, PR, and HER2 were previously determined [16,17]. Outcome data included survival status, survival time, cause of death, development, and time to locoregional recurrence and distant metastasis (DM). BC-specific survival (BCSS) is defined as the time (in months) from the date of primary surgery to the date of a BC-related death. Disease-free survival (DFS) is defined as the time (in months) from the date of primary surgery to the appearance of recurrence. Distant metastasis-free survival (DMFS) is defined as the time (in months) from the date of primary surgery to the appearance of DM. Treatments include chemotherapy (cyclophosphamide, methotrexate, and fluorouracil (CMF)) or endocrine therapy.

### 2.4. Evaluation of TRIM2 Protein Expression Using Immunohistochemistry 

Prior to IHC staining, the specificity of the TRIM2 antibody (1:1000, 67342-1-Ig, Proteintech, Manchester, UK) was validated using Western blotting and fluorescent secondary antibodies: (IR Dye 800CW donkey anti-rabbit and 680RD donkey anti-mouse at 1:15,000 (LI-COR Biosciences, Cambridge, UK). TRIM2 was investigated in human ER+ (MCF-7, ZR-75-1, HCC1500) and TN (MDA-MB-231, MDA-MB-436, and MDA-MB-468) BC cell lysates (American Type Culture Collection; Rockville, MD, USA), which showed two bands at the predicted size of approximately 72 and 80 kDa) in MCF-7 and just the 72 kDa band in other ER+ and TN cell lines (Supplementary Figure S1). The mouse anti-β-actin antibody (A5441, Clone AC-15; Sigma-Aldrich, Gillingham, UK) was used at 1:5000 as a housekeeping protein and showed a band at approximately 42 kDa.

TRIM2 protein expression was evaluated using IHC on 4 μm TMA sections using the Novolink polymer detection system (RE7150-K, Leica Biosystems, Milton Keynes, UK), according to manufacturer instructions and as previously described [17]. Ten full-face BC tissue sections from the cohort were additionally stained and assessed for heterogeneity by a pathologist (AI). Heat-induced antigen epitope retrieval was performed in a citrate buffer (pH 6.0) for 20 min using a microwave oven (JT359 Jet Chef 1000 W, Whirlpool UK Appliances Limited, Peterborough, UK). Tissues were incubated with a TRIM2 monoclonal antibody (67342-1-Ig, Proteintech, Manchester, UK) at 1:500 in antibody diluent (RE AR9352, Leica Biosystems, Newcastle upon Tyne, UK) at room temperature for 90 min. Negative (omission of the primary antibody) and positive (liver tissue) controls were included according to the manufacturer’s datasheet. 

### 2.5. Immunohistochemical Scoring

A Nanozoomer scanner (Hamamatsu Photonics, Welwyn Garden City, UK) was used to scan stained sections as high-resolution digital images at 20× magnification, viewed using Xplore software version 5.3.0 (Phillips Healthcare, Belfast, UK). Blind double scoring was performed by two researchers (BKM and AF) to evaluate inter-observer concordance. Interclass correlation coefficient (ICC) concordance showed good reliability between both observers (0.721). TRIM2 protein expression was assessed using a modified histochemical score (H-score) and dichotomised into low (≤35 H-score) and high (>35 H-score) expression derived from the prediction of patient survival using X-tile version 3.6.1 (https://medicine.yale.edu/lab/rimm/research/software.aspx; Yale University).

### 2.6. Statistical Analysis

SPSS (version 25 Chicago, IL, USA) was used to perform statistical analysis. A chi-square test was used to evaluate the association between *TRIM2* mRNA/TRIM2 protein expression and clinicopathological parameters. To test the correlation between two continuous normalised data, Pearson’s correlation coefficient was used. Differences in the mean between three or more groups were assessed using one-way analysis of variance (ANOVA) with a post-hoc Tukey multiple comparison test (for normalised data), while Mann–Whitney and Kruskal–Wallis tests were applied for non-parametric data. The chi-square test (*x*^2^) was performed for inter-relationships between categorical variables, including associations with clinicopathological parameters and other biological markers. Kaplan–Meier survival curves and a log-rank test were used to assess the association between *TRIM2* mRNA/TRIM2 protein expression and clinical outcomes. Cox regression analysis was used to evaluate the independent prognostic significance of TRIM2 expression. *p* values were adjusted using Bonferroni correction for multiple comparisons. A *p* value < 0.05 for all the tests was considered significant.

## 3. Results

### 3.1. Glutaminase-Related Signalling Pathways 

**Figure 1:**
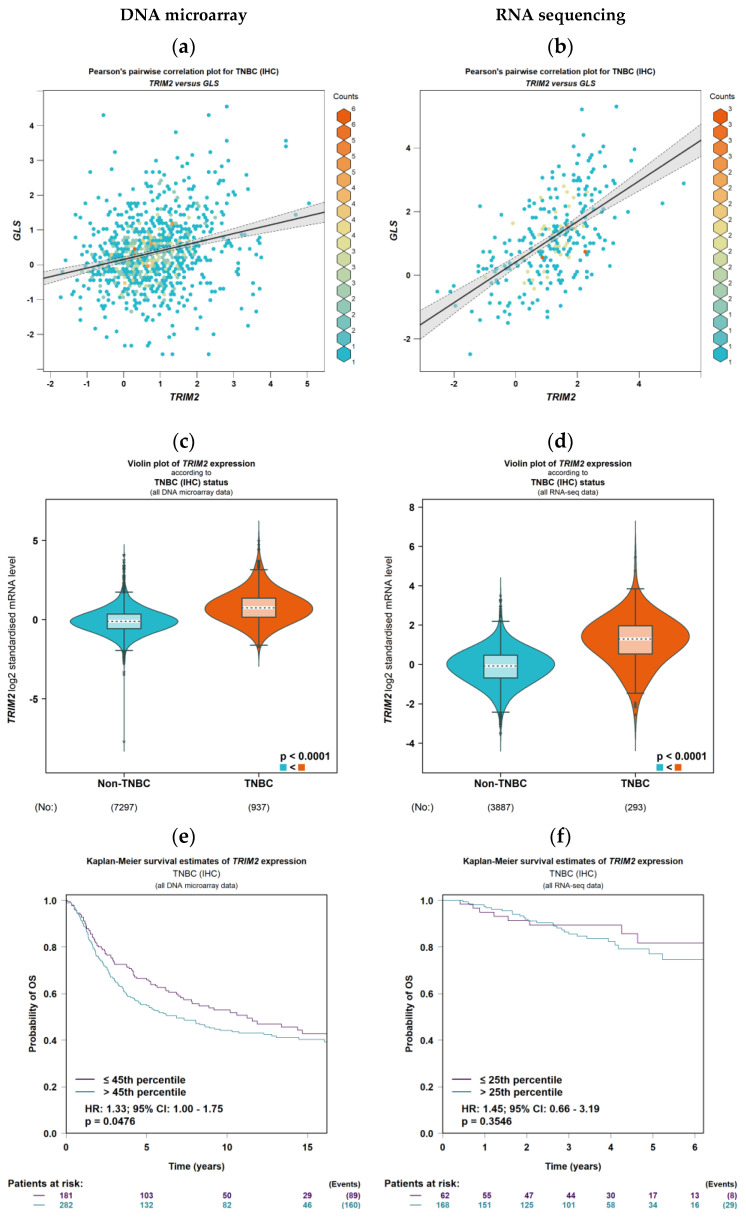
*TRIM2* mRNA expression and patient outcome in triple-negative breast cancer using DNA microarray and RNA sequencing datasets within bc-GenExMiner: (**a**,**b**) correlation between *TRIM2* and *GLS* mRNA expression, (**c**,**d**) *TRIM2* mRNA according to triple-negative breast cancer status, (**e**,**f**) Kaplan-Meier overall survival estimates of *TRIM2* mRNA expression.

In BC, *TRIM2* emerged as the most significant gene linked to *GLS* expression, standing out among nine other identified candidates based on the Z-score and *p* value (Table 1). Analysis across both DNA microarray and RNA sequencing data solidified the link between *TRIM2* and *GLS* expression in all patients, with an even stronger association observed in ER-negative tumours and TNBC (Figure 1a,b and Supplementary Figure S2).

### 3.2. TRIM2 Gene Expression in Breast Cancer 

Elevated *TRIM2* mRNA levels strongly correlated with ER-negative and TNBC in both DNA microarray and RNA sequencing analyses (*p* < 0.0001, Figure 1c,d and Supplementary Figure S3a,b).

The impact of *TRIM2* mRNA expression on OS was complex and dependent on ER status. While no overall association was observed (Supplementary Figure S3c), high *TRIM2* levels were significantly linked to worse OS in TNBC and ER-negative tumours (both *p* < 0.05, Figure 1e and Supplementary Figure S3g). Interestingly, the opposite trend emerged in ER-positive tumours, where high *TRIM2* mRNA correlated with longer OS (*p* < 0.0001, Supplementary Figure S3e). Even when accounting for established prognostic factors such as tumour size, tumour grade, and lymph node stage, high *TRIM2* mRNA expression remained a powerful predictor of BCSS (*p* < 0.01, Supplementary Table S2), reinforcing its potential as an independent prognostic marker.

These results hint at a complex interplay between *TRIM2* and BC, particularly across different subtypes. To unravel its full story, further investigation of TRIM2 protein expression in a large cohort of BCs was therefore warranted. 

### 3.3. TRIM2 Protein Expression in Breast Cancer 

Analysis of full-face BC tissue sections confirmed the suitability of using TMAs for studying TRIM2 protein expression. The protein was found exclusively in the cytoplasm of invasive tumour cells, with a spectrum of intensity ranging from absent to high. Representative images of TRIM2 protein expression in BC are shown in Supplementary Figure S1. Notably, high TRIM2 expression was detected in nearly half of the cases (47%, 353/749). 

Consistent with mRNA results, high TRIM2 protein expression showed a significant association with ER-negative tumours and TNBC (both *p* < 0.0001, Table 2). TRIM2 also displayed a curious association with low tumour grade (*p* < 0.0001, Table 2), while remaining unrelated to other clinicopathological parameters including tumour size, nodal stage, and HER2 status.

### 3.4. Association of TRIM2 Expression with Patient Outcome

**Figure 2:**
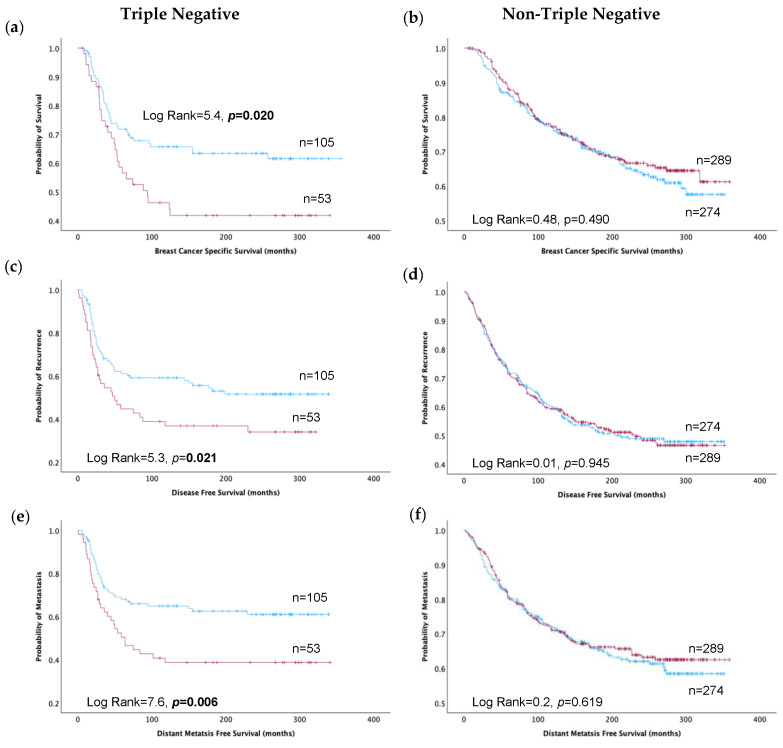
Kaplan–Meier estimates depicting TRIM2 high (red line) and TRIM2 low (blue line) protein expression and survival outcomes in triple-negative and non-triple-negative breast cancer: (**a**,**b**) Breast cancer-specific survival. (**c**,**d**) Disease-free survival. (**e**,**f**) Distant metastasis-free survival.

High TRIM2 protein levels emerged as a strong predictor of poor outcomes in TNBC and ER-negative patients, significantly impacting BCSS (*p* < 0.05), DFS (*p* < 0.05), and DMFS (*p* < 0.01) (Figure 2 and Supplementary Figure S5). Notably, this association was absent in all cases, including ER-positive and non-TNBC patients (all *p* > 0.05, Figure 2, Supplementary Figures S4 and S5).

**Figure 3:**
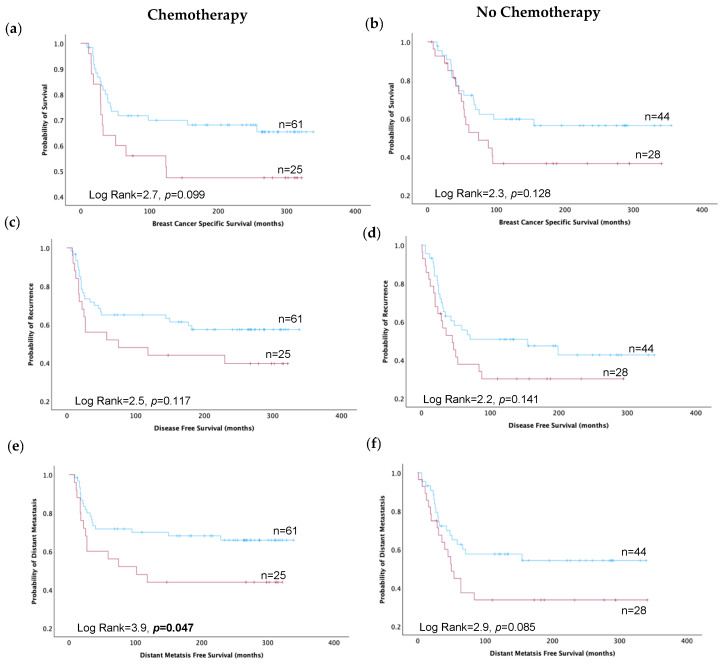
Kaplan–Meier estimates depicting TRIM2 high (red line) and TRIM2 low (blue line) protein expression and survival outcomes in triple-negative breast cancer patients treated with or without chemotherapy: (**a**,**b**) Breast cancer-specific survival. (**c**,**d**) Disease-free survival. (**e**,**f**) Distant metastasis-free survival.

TRIM2 held predictive power for DMFS in all patients receiving chemotherapy (*p* < 0.05, Supplementary Figure S6e), particularly in TNBC (*p* < 0.05, Figure 3e) and ER-negative tumours (*p* < 0.01, Supplementary Figure S8). Its influence on BCSS and DFS was absent, regardless of chemotherapy or tumour type (all *p* > 0.05, Figure 3a,c and Supplementary Figure S6a,c) except in ER-negative tumours (*p* < 0.01, Supplementary Figure S8a,c). Likewise, no significant association between TRIM2 expression and patient outcome was observed for ER-negative and non-TNBC patients, regardless of chemotherapy regimens (all *p* > 0.05, Supplementary Figures S7 and S9).

In both TNBC and ER-negative tumours, high TRIM2 protein retained its predictive power for BCSS, DFS, and DMFS, remaining independent of established prognostic factors, namely, tumour size, tumour grade, and lymph node stage (*p* < 0.05, Table 3 and Supplementary Table S3). Notably, this association was absent in non-TNBC and ER-positive tumours (*p* > 0.05).

## 4. Discussion

BC is a complex disease with high incidence rates worldwide and has the second-highest mortality rate in women among all cancers [18]. The disease is classified into five intrinsic subtypes that require different treatment strategies, and it still remains heterogeneous in terms of its prognosis and response to different treatment options [3]. TNBC is an aggressive form of BC that accounts for 10–15% of all breast cancer subtypes. Discovering new drivers within the stratified BC subtypes with potential as novel therapeutic targets is of urgent need.

This study explored the functional role of GLS and potential genes that interact with *GLS* using gene pathway analysis and identified *TRIM2* as an important gene co-expressed with GLS. There is no direct evidence that *TRIM2* directly interacts with, or regulates, *GLS*. However, in TNBC cells, the depletion of eEF2K leads to an increase in TRIM2. Interestingly, eEF2K depletion, combined with either depriving cells of glutamine or inhibiting GLS, further suppresses the growth [19].

Altered metabolic enzymes can drive cancer progression, and deregulated cancer metabolism has gained attention in recent years and is regarded as a new hallmark of cancer [4,6]. This study provides evidence at both mRNA and protein levels that TRIM2 is a promising biomarker influencing patient outcomes. This is the first study to provide evidence on the potential of TRIM2 protein as a new prognostic biomarker for BC.

It has been reported that TRIM2 plays an oncogenic role in cancer types including lung adenocarcinoma and colorectal cancer and has prognostic value [20,21]. However, in BC, there are limited reports of TRIM2 and its prognostic effect. Previous evidence has recognised E3 ubiquitin ligase as an important carcinogenesis regulator and suggests that some ubiquitinases promote tumorigenesis by regulating the ubiquitination level of tumour suppressors or carcinogenic substrates [22,23,24,25,26]. Previous studies have shown that the TRIM family members play important roles in regulating biological processes including cell growth, differentiation, development, apoptosis, inflammation, and immunity. TRIM2, a key member of the TRIM family, plays an important role in both malignant and non-malignant diseases. TRIM2 is considered an oncogene, as it is highly expressed in many cancers and related to tumour cell proliferation, apoptosis, metastasis, and angiogenesis [20,22,23]. In a study to evaluate ovarian cancer progression, high TRIM2 expression promoted proliferation and invasion in ovarian cancer cells [27]. TRIM2 also regulated the metastasis of colorectal cancer cells through EMT in vivo and in vitro [20]. Moreover *TRIM2* knockdown significantly reduced the proliferation, colony formation, migration, and invasion of lung adenocarcinoma cells [21]. In BC, TRIM2 is highly expressed in the tamoxifen-resistant BC cell line MCF-7R and has thus been linked with tamoxifen resistance [23].

The present study used large well-characterised cohorts of clinically annotated patients with primary BC to explore the prognostic value of TRIM2 at genomic and proteomic levels. This study showed that the expression of TRIM2 in BC was associated with poor prognosis and patient survival independent of other clinicopathological variables. The findings demonstrated that high TRIM2 expression is associated with poor clinicopathological features. TRIM2 in osteosarcoma reduces Bcl-2-interacting mediator (Bim) expression and causes excessive proliferation of cancer cells via the PI3K/AKT/mTOR signalling pathway. The study indicated that the PI3K/AKT pathway may be involved in regulating TRIM2 in the development and metastasis of osteosarcoma tumour cells [22]. mTORC1 is one of the PI3K family members that is important in the regulation of the cell cycle, as well as in growth and development; moreover, it is involved in the proliferation, migration, and survival of some cancers such as breast, osteosarcoma, pancreatic, and cervical [28]. The results in this study are consistent with other studies in colorectal cancer and osteosarcoma, which similarly showed that high TRIM2 expression was associated with unfavourable clinical outcomes and metastasis promotion [22]. However, some previous reports showed opposite results with the expression of TRIM2, which is downregulated in clear cell renal cell carcinoma, affecting cell proliferation and migration, and also showed opposite results regarding patients’ survival [29]. Previous studies have suggested the presence of glutamine and subsequent glutaminolysis to be implicated in the mTORC1 signalling pathway [30,31]. This suggests that TRIM2 may be involved in the pathogenesis of cancer via different mechanisms, although the underlying mechanism may be complex and diverse, requiring further validation.

Regarding BC molecular subtypes and prognostic significance in BC, the expression of TRIM2 was significantly correlated with TNBC. This was consistent with outcome analysis, which showed poor patient survival in TNBC. Few studies have investigated the relationship between TRIM2 and BC; moreover, to date, none have demonstrated such findings. Another striking finding in the current study is that high TRIM2 expression was associated with worse OS in patients stratified according to chemotherapy treatment. Overall, this study supports the involvement of TRIM2 and glutamine metabolism as factors affecting BC progression and, ultimately, patient outcome through involvement in upstream signalling pathways. To our understanding, this study is the first to show this relationship. However, it is important to note that functional studies are warranted to help elucidate the role of TRIM2 in BC and the mechanism involved. In vivo xenograft mouse model studies could determine the upregulation effects of TRIM2 involved in tumour growth. Similarly, knockdown studies of *TRIM2* using in vitro models are needed to explore the regulatory mechanisms of TRIM2 in tumorigenesis. Notably, to date, there have been no TRIM2 inhibitors made commercially available for cancer therapy.

## 5. Conclusions

In summary, this study provides evidence that the high expression of TRIM2 in BC is related to short patient survival and has potential as a novel biomarker of patient therapy response. This study has identified a previously unrecognised role of TRIM2 in BC biology and behaviour, as well as its potential role as a prognostic biomarker. Further validation through in vivo and in vitro analysis using BC models to investigate whether TRIM2 expression impacts cancer cell proliferation, invasion, and the underlying mechanisms of development and progression is warranted.

## Figures and Tables

**Table 1:** Top 10 genes co-expressed with *GLS* ranked based on Z-score and *p* value.

Gene	Z-Score	*p* Value
*TRIM2*	0.60	**9.9 × 10^−88^**
*ARHGAP21*	0.56	**1.0 × 10^−88^**
*CHST3*	0.56	**1.2 × 10^−88^**
*DOCK7*	0.55	**5.2 × 10^−88^**
*FOXN2*	0.54	**2.9 × 10^−87^**
*MTMR2*	0.54	**6.6 × 10^−82^**
*CLIP4*	0.53	**1.2 × 10^−78^**
*MRAS*	0.53	**3.5 × 10^−78^**
*MAML2*	0.53	**4.5 × 10^−78^**

*p* values in bold are statistically significant.

**Table 2:** Clinicopathological association of TRIM2 protein in breast cancer.

Parameters	Low TRIM2 *n* (%)	High TRIM2 *n* (%)	*p* Value
Tumour size			0.211
<2 cm	176 (50)	173 (50)	
≥2 cm	220 (55)	180 (45)	
Tumour grade			**0.00007**
1	43 (44)	55 (56)	
2	105 (44)	133 (56)	
3	248 (60)	165 (40)	
Lymph Node Stage			0.699
1	238 (54)	204 (46)	
2	124 (52)	113 (48)	
3	34 (49)	36 (51.4)	
Vascular Invasion			0.472
Negative	248 (52)	148 (55)	
Positive	230 (48)	123 (45)	
Histological subtypes			0.217
Ductal no-special type	269 (55)	217 (45)	
Lobular	35 (53)	31 (47)	
Metaplastic carcinoma	3 (75)	1 (25)	
Other special type	17 (50)	17 (50)	
Mixed NST and other special type	72 (45)	87 (55)	
Estrogen Receptor			
Negative	147 (64)	82 (36)	**0.00003**
Positive	247 (48)	270 (52)	
Progesterone Receptor			
Negative	184 (57)	137 (43)	0.096
Positive	199 (49)	205 (51)	
HER2			
Negative	325 (53)	289 (47)	0.635
Positive	54 (55)	44 (45)	
Triple Negative			
No	274 (49)	289 (51)	**0.0003**
Yes	105 (61)	11 (39)	

*p* values in bold are statistically significant.

**Table 3:** Multivariate survival analysis of prognostic parameters and TRIM2 protein expression in relation to patient outcome using Cox-regression in triple-negative breast cancer.

	Triple Negative		Non-Triple Negative	
Parameters	Hazard Ratio (95% CI)	*p* Value	Hazard Ratio (95% CI)	*p* Value
Breast Cancer-Specific Survival				
TRIM2 protein	1.6 (1.0–2.7)	**0.048**	1.0 (0.7–1.3)	0.865
Tumour Size	1.6 (0.9–2.8)	0.108	1.8 (1.3–2.5)	**0.0004**
Grade	1.2 (0.7–2.1)	0.524	1.5 (1.2–1.9)	**0.0003**
Nodal stage	1.8 (1.3–2.5)	**0.0003**	2.1 (1.7–2.7)	**2.8 × 10^−11^**
Disease-Free Survival				
TRIM2 protein	1.6 (1.0–2.5)	**0.033**	1.1 (0.8–1.3)	0.639
Tumour Size	1.5 (0.9–2.4)	0.121	1.3 (1.0–1.7)	**0.027**
Grade	1.2 (0.5–2.6)	0.714	1.2 (1.0–1.5)	**0.019**
Nodal stage	1.6 (1.2–2.1)	**0.003**	1.7 (1.4–2.1)	**5.7 × 10^−9^**
Distant Metastasis-Free Survival				
TRIM2 protein	1.8 (1.1–2.9)	**0.015**	1.0 (0.7–1.3)	0.957
Tumour size	1.4 (0.8–2.3)	0.266	2.0 (1.4–2.7)	**0.00003**
Grade	1.0 (0.4–2.3)	0.979	1.4 (1.1–1.8)	**0.002**
Nodal stage	1.8 (1.3–2.5)	**0.0002**	2.0 (1.6–2.5)	**8.3 × 10^−11^**

*p* values in bold are statistically significant.

## Data Availability

The data presented in the current study are available upon reasonable request.

## Supplementary Materials

The following supporting information can be downloaded at: https://www.mdpi.com/article/10.3390/cancers16111949/s1, Supplementary Figure S1. TRIM2 protein expression. (a) Western blot analysis showing TRIM2 protein expression in breast cancer cell lines at the correct predicted sizes of 80 kDa and 72 kDa, β-actin was included as a positive control, (b) TRIM2 protein in invasive breast cancer using immunohistochemistry showing low, moderate and high expression. Magnification 10×. Scale bars = 100 μm; Supplementary Figure S2. Correlation between TRIM2 and GLS mRNA expression in breast cancer using DNA microarray and RNA sequencing datasets within bc-GenExMiner: (a,b) all patients, (c,d) Estrogen Receptor positive tumours, (e,f) Estrogen Receptor negative tumours; Supplementary Figure S3. TRIM2 mRNA expression and patient outcome in estrogen receptor positive and estrogen receptor negative breast cancer using DNA microarray and RNA sequencing datasets within bc-GenExMiner: (a,b) TRIM2 mRNA according to Estrogen Receptor status, (c,d) Kaplan-Meier overall survival estimates of TRIM2 mRNA expression in all cases, (e,f) Kaplan-Meier overall survival estimates of TRIM2 mRNA expression in Estrogen Receptor positive tumours, (g,h) Kaplan-Meier overall survival estimates of TRIM2 mRNA expression in Estrogen Receptor negative tumours; Supplementary Figure S4. Kaplan-Meier estimates of TRIM2 protein expression in breast cancer: (a) Breast Cancer Specific Survival, (b) Disease Free Survival, (c) Distant Metastasis Free Survival. TRIM2 high (red line) and low (blue line); Supplementary Figure S5. Kaplan-Meier estimates of TRIM2 protein expression in Estrogen Receptor positive and Estrogen Receptor negative breast cancer: (a,b) Breast Cancer Specific Survival, (c,d) Disease Free Survival, (e,f) Distant Metastasis Free Survival. TRIM2 high (red line) and low (blue line); Supplementary Figure S6. Kaplan-Meier estimates of TRIM2 protein expression in breast cancer treated with or without chemotherapy: (a,b) Breast Cancer Specific Survival, (c,d) Disease Free Survival, (e,f) Distant Metastasis Free Survival. TRIM2 high (red line) and low (blue line); Supplementary Figure S7. Kaplan-Meier estimates of TRIM2 protein expression in Estrogen Receptor positive breast cancer treated with or without chemotherapy: (a,b) Breast Cancer Specific Survival, (c,d) Disease Free Survival, (e,f) Distant Metastasis Free Survival. TRIM2 high (red line) and low (blue line); Supplementary Figure S8. Kaplan-Meier estimates of TRIM2 protein expression in Estrogen Receptor negative breast cancer treated with or without chemotherapy: (a,b) Breast Cancer Specific Survival, (c,d) Disease Free Survival, (e,f) Distant Metastasis Free Survival. TRIM2 high (red line) and low (blue line); Supplementary Figure S9. Kaplan-Meier estimates of TRIM2 protein expression in non-triple negative breast cancer treated with or without chemotherapy: (a,b) Breast Cancer Specific Survival, (c,d) Disease Free Survival, (e,f) Distant Metastasis Free Survival. TRIM2 high (red line) and low (blue line); Supplementary Table S1. Patient and clinicopathological parameters of the Nottingham breast cancer cohort; Supplementary Table S2. Multivariate survival analysis of prognostic parameters and TRIM2 mRNA and TRIM2 protein expression in relation to patient outcome using Cox-regression; Supplementary Table S3. Multivariate survival analysis of prognostic parameters and TRIM2 protein expression in relation to patient outcome using Cox-regression in Estrogen Receptor Breast Cancer.
