# Supplementary material for: Tripartite Motif-Containing 2, a Glutamine Metabolism-Associated Protein, Predicts Poor Patient Outcome in Triple-Negative Breast Cancer Treated with Chemotherapy
Source: Cancers (Basel). 2024 May 21;16(11):1949. doi: 10.3390/cancers16111949 (PMC11171213; doi:10.3390/cancers16111949)
Supplement: Supplementary file 1 [file cancers-16-01949-s001.zip › cancers-2854957-supplementary.pdf]

Supplementary Table 1. Patient and clinicopathological parameters of the Nottingham breast cancer cohort

Parameters	Number	Percentage
**Age**		
<50	277	37
≥50	472	63
**Referral**		
Screening	174	23
Symptomatic	575	77
**Tumour size**		
<2 cm	349	47
≥2 cm	400	53
**Tumour grade**		
1	98	13
2	238	32
3	413	55
**Lymph Node Stage**		
1	442	59
2	237	32
3	70	9
**Vascular Invasion**		
Negative	478	64
Positive	271	36
**Histological subtypes**		
Ductal no-special type	486	65
Lobular	66	9
Metaplastic carcinoma	4	1
Other special type	34	5
Mixed NST and other special type	159	21
**Estrogen Receptor**		
Negative	229	31
Positive	517	69
**Progesterone Receptor**		
Negative	321	44
Positive	404	56
**HER2**		
Negative	614	86
Positive	98	14
**Triple Negative**		
No	563	78
Yes	185	22
**Chemotherapy**		
No	564	75
Yes	25	25
**Follow-up**	5-358 months (mean 173 months)	
**Status**		
Alive	316	42
Died from breast cancer	261	35
Died from other causes	172	23

Supplementary Table 2. Multivariate survival analysis of prognostic parameters and *TRIM2* mRNA and TRIM2 protein expression in relation to patient outcome using Cox-regression

	***TRIM2* mRNA**		**TRIM2 protein**	
**Parameters**	**Hazard ratio (95 % CI)**	***p* value**	**Hazard ratio (95 % CI)**	***p* value**
TRIM2	1.4 (1.1-1.8)	**0.003**	1.1 (1.0-1.7)	0.273
Tumour size	1.7 (1.2-2.3)	**<0.001**	1.7 (1.3-2.1)	**0.0002**
Grade	1.9 (1.5-2.4)	**<0.0001**	1.5 (1.2-1.8)	**0.00009**
Nodal stage	2.9 (1.7-2.3)	**<0.0001**	2.0 (1.7-2.4)	**$2.4x10^{-14}$**
**Disease Free Survival**				
TRIM2			1.2 (1.0-1.4)	0.132
Tumour Size			1.3 (1.0-1.7)	**0.012**
Grade	Not available		1.2 (1.0-1.4)	**0.017**
Nodal stage			1.7 (1.5-2.0)	**$1.8x10^{-11}$**
**Distant Metastasis Free Survival**				
TRIM2			1.0 (0.7-1.3)	0.748
Tumour size			2.0 (1.5-2.8)	**0.00003**
Grade	Not available		1.4 (1.2-1.8)	**0.001**
Nodal stage			2.0 (1.6-2.5)	**$3.0x10^{-10}$**

p values in bold denote statistically significant.

Supplementary Table 3. Multivariate survival analysis of prognostic parameters and TRIM2 protein expression in relation to patient outcome using Cox-regression in Estrogen Receptor Breast Cancer.

	**ER-**		**ER+**	
**Parameters**	**Hazard ratio (95 % CI)**	**p value**	**Hazard ratio (95 % CI)**	**p value**
**Breast Cancer Specific Survival**				
TRIM2	1.7 (1.1-2.6)	**0.013**	0.9 (0.7-1.2)	0.580
Tumour Size	1.5 (0.9-2.3)	0.101	1.8 (1.3-2.5)	**0.0007**
Grade	1.2 (0.7-2.0)	0.593	1.6 (1.2-2.0)	**0.0002**
Nodal stage	1.8 (1.3-2.3)	**0.00009**	2.1 (1.7-2.7)	**$6.1x10^{-11}$**
**Disease Free Survival**				
TRIM2	1.7 (1.1-2.4)	**0.007**	1.0 (0.8-1.3)	0.996
Tumour Size	1.2 (0.8-1.8)	0.342	1.4 (1.1-1.8)	**0.016**
Grade	1.0 (0.7-1.6)	0.905	1.2 (1.0-1.5)	**0.020**
Nodal stage	1.6 (1.2-2.0)	**0.0006**	1.8 (1.4-2.1)	**$1.1x10^{-8}$**
**Distant Metastasis Free Survival**				
TRIM2	1.7 (1.1-2.5)	**0.011**	1.0 (0.7-1.3)	0.748
Tumour size	1.3 (0.8-2.0)	0.274	2.0 (1.5-2.8)	**0.00003**
Grade	1.1 (0.7-1.8)	0.684	1.4 (1.2-1.8)	**0.001**
Nodal stage	1.8 (1.4-2.4)	**0.00002**	2.0 (1.6-2.5)	**$3.0x10^{-10}$**

p values in bold denote statistically significant.

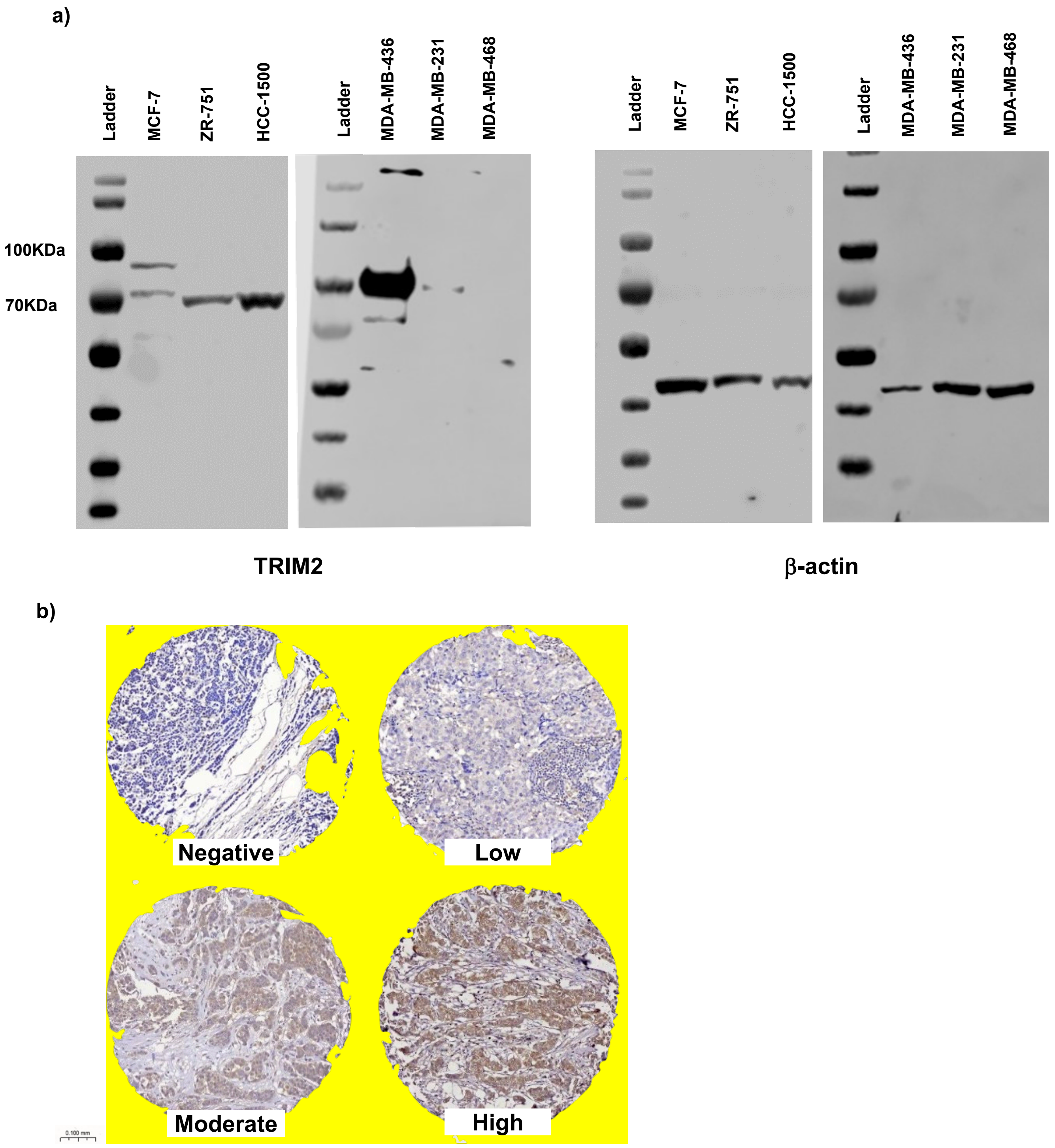
a)
Ladder
MCF-7
ZR-751
HCC-1500
Ladder
MDA-MB-436
MDA-MB-231
MDA-MB-468
Ladder
MCF-7
ZR-751
HCC-1500
Ladder
MDA-MB-436
MDA-MB-231
MDA-MB-468
100KDa
70KDa
TRIM2
β-actin
b)
Negative
Low
Moderate
High

Supplementary Figure 1. TRIM2 protein expression. a) Western blot analysis showing TRIM2 protein expression in breast cancer cell lines at the correct predicted sizes of 80kDa and 72kDa, β-actin was included as a positive control, b) TRIM2 protein in invasive breast cancer using immunohistochemistry showing low, moderate and high expression. Magnification 10x. Scale bars=100μm.

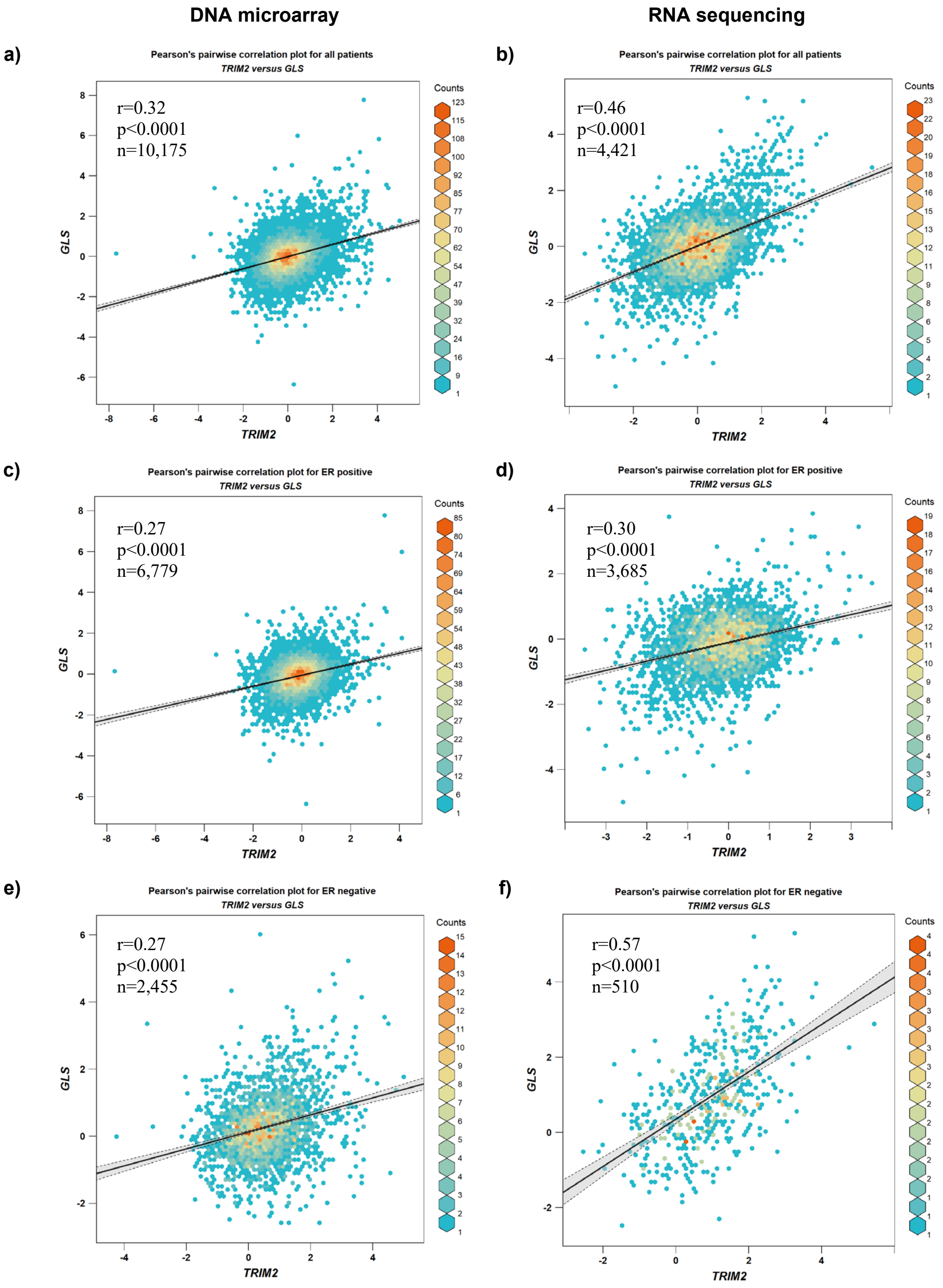

Supplementary Figure 2. Correlation between *TRIM2* and *GLS* mRNA expression in breast cancer using DNA microarray and RNA sequencing datasets within bc-GenExMiner: a-b) all patients, c-d) Estrogen Receptor positive tumours, e-f) Estrogen Receptor negative tumours

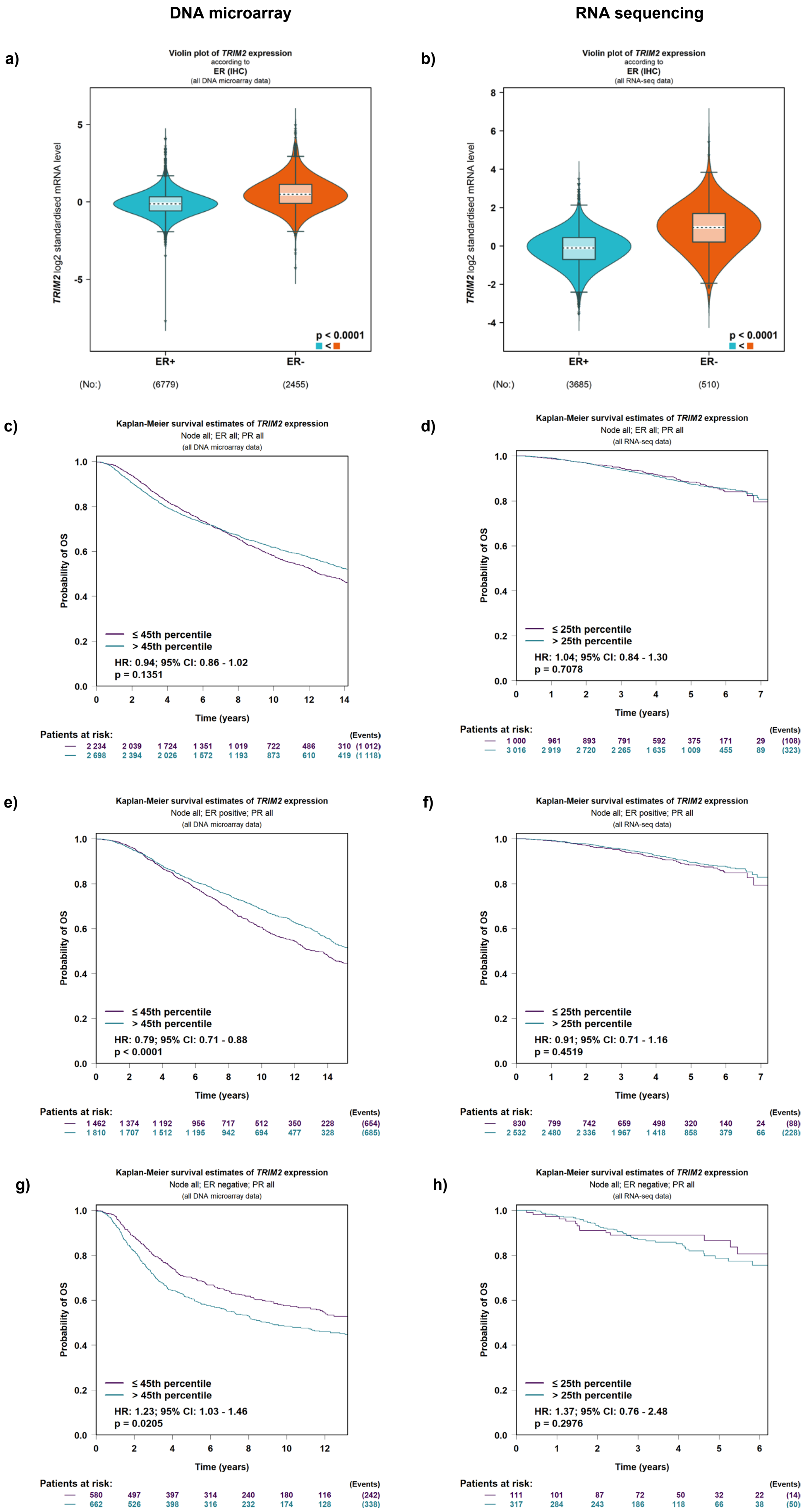
DNA microarray
RNA sequencing
a)
Violin plot of TRIM2 expression
according to
ER (IHC)
(all DNA microarray data)
TRIM2 log2 standardised mRNA level
p < 0.0001
ER+
ER-
(No:)
(6779)
(2455)
b)
Violin plot of TRIM2 expression
according to
ER (IHC)
(all RNA-seq data)
TRIM2 log2 standardised mRNA level
p < 0.0001
ER+
ER-
(No:)
(3685)
(510)
c)
Kaplan-Meier survival estimates of TRIM2 expression
Node all; ER all; PR all
(all DNA microarray data)
Probability of OS
≤ 45th percentile
> 45th percentile
HR: 0.94; 95% CI: 0.86 - 1.02
p = 0.1351
Time (years)
Patients at risk:
(Events)
2 234 2 039 1 724 1 351 1 019 722 486 310 (1 012)
2 698 2 394 2 026 1 572 1 193 873 610 419 (1 118)
d)
Kaplan-Meier survival estimates of TRIM2 expression
Node all; ER all; PR all
(all RNA-seq data)
Probability of OS
≤ 25th percentile
> 25th percentile
HR: 1.04; 95% CI: 0.84 - 1.30
p = 0.7078
Time (years)
Patients at risk:
(Events)
1 000 961 893 791 592 375 171 29 (108)
3 016 2 919 2 720 2 265 1 635 1 009 455 89 (323)
e)
Kaplan-Meier survival estimates of TRIM2 expression
Node all; ER positive; PR all
(all DNA microarray data)
Probability of OS
≤ 45th percentile
> 45th percentile
HR: 0.79; 95% CI: 0.71 - 0.88
p < 0.0001
Time (years)
Patients at risk:
(Events)
1 462 1 374 1 192 956 717 512 350 228 (654)
1 810 1 707 1 512 1 195 942 694 477 328 (685)
f)
Kaplan-Meier survival estimates of TRIM2 expression
Node all; ER positive; PR all
(all RNA-seq data)
Probability of OS
≤ 25th percentile
> 25th percentile
HR: 0.91; 95% CI: 0.71 - 1.16
p = 0.4519
Time (years)
Patients at risk:
(Events)
830 799 742 659 498 320 140 24 (88)
2 532 2 480 2 336 1 967 1 418 858 379 66 (228)
g)
Kaplan-Meier survival estimates of TRIM2 expression
Node all; ER negative; PR all
(all DNA microarray data)
Probability of OS
≤ 45th percentile
> 45th percentile
HR: 1.23; 95% CI: 1.03 - 1.46
p = 0.0205
Time (years)
Patients at risk:
(Events)
580 497 397 314 240 180 116 (242)
662 526 398 316 232 174 128 (338)
h)
Kaplan-Meier survival estimates of TRIM2 expression
Node all; ER negative; PR all
(all RNA-seq data)
Probability of OS
≤ 25th percentile
> 25th percentile
HR: 1.37; 95% CI: 0.76 - 2.48
p = 0.2976
Time (years)
Patients at risk:
(Events)
111 101 87 72 50 32 22 (14)
317 284 243 186 118 66 38 (50)

Supplementary Figure 3. *TRIM2* mRNA expression and patient outcome in estrogen receptor positive and estrogen receptor negative breast cancer using DNA microarray and RNA sequencing datasets within bc-GenExMiner: a-b) *TRIM2* mRNA according to Estrogen Receptor status, c-d) Kaplan-Meier overall survival estimates of *TRIM2* mRNA expression in all cases, e-f) Kaplan-Meier overall survival estimates of *TRIM2* mRNA expression in Estrogen Receptor positive tumours, g-h) Kaplan-Meier overall survival estimates of *TRIM2* mRNA expression in Estrogen Receptor negative tumours

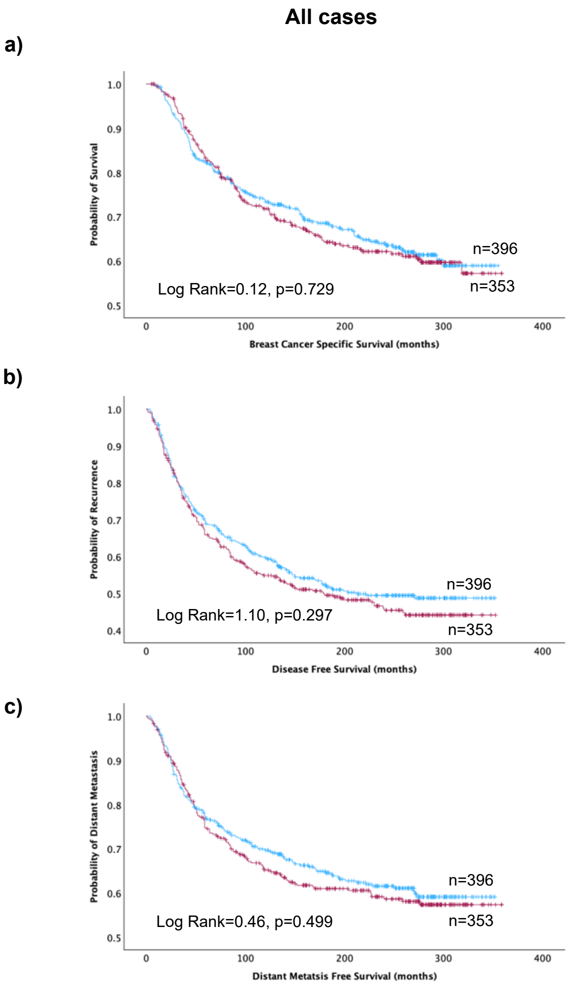

Supplementary Figure 4. Kaplan-Meier estimates of TRIM2 protein expression in breast cancer: a) Breast Cancer Specific Survival, b) Disease Free Survival, c) Distant Metastasis Free Survival. TRIM2 high (red line) and low (blue line)

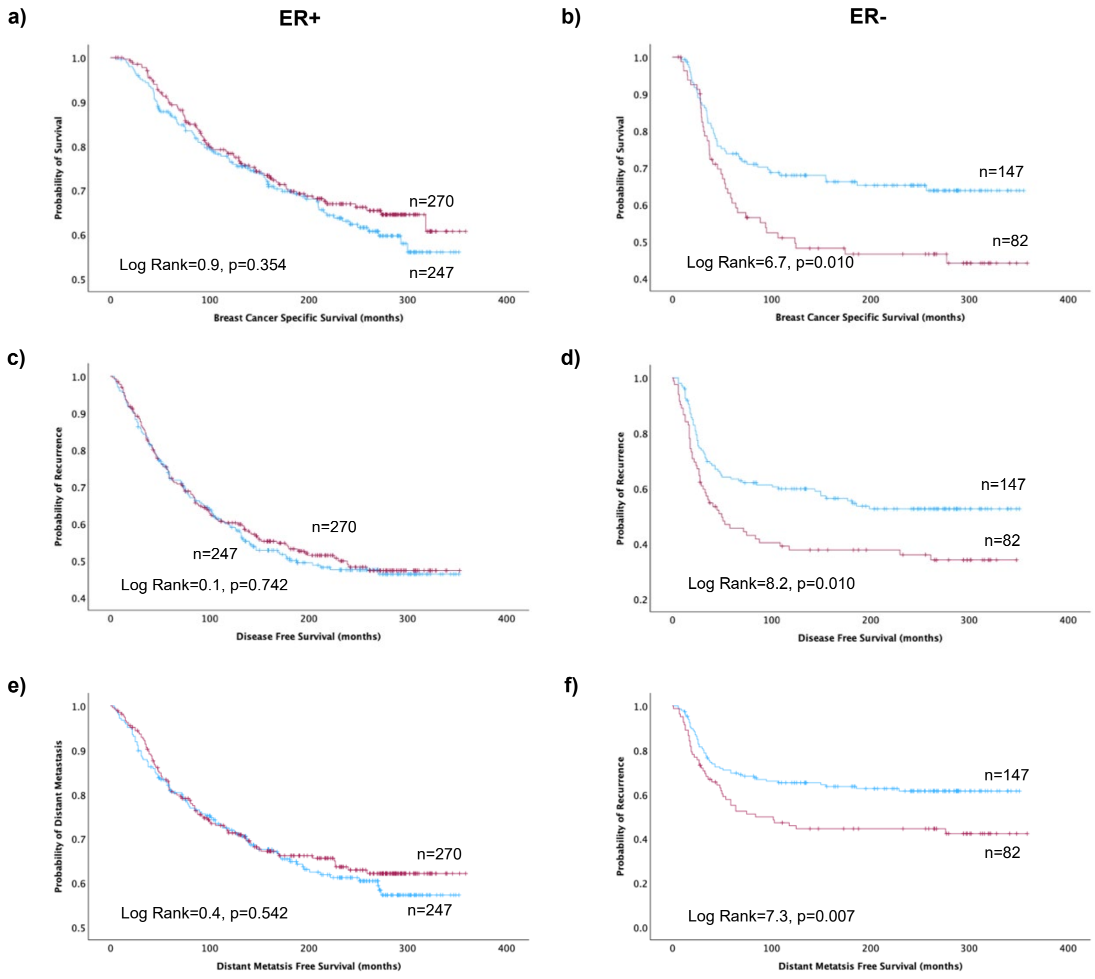

Supplementary Figure 5. Kaplan-Meier estimates of TRIM2 protein expression in Estrogen Receptor positive and Estrogen Receptor negative breast cancer: a-b) Breast Cancer Specific Survival, c-d) Disease Free Survival, e-f) Distant Metastasis Free Survival. TRIM2 high (red line) and low (blue line)

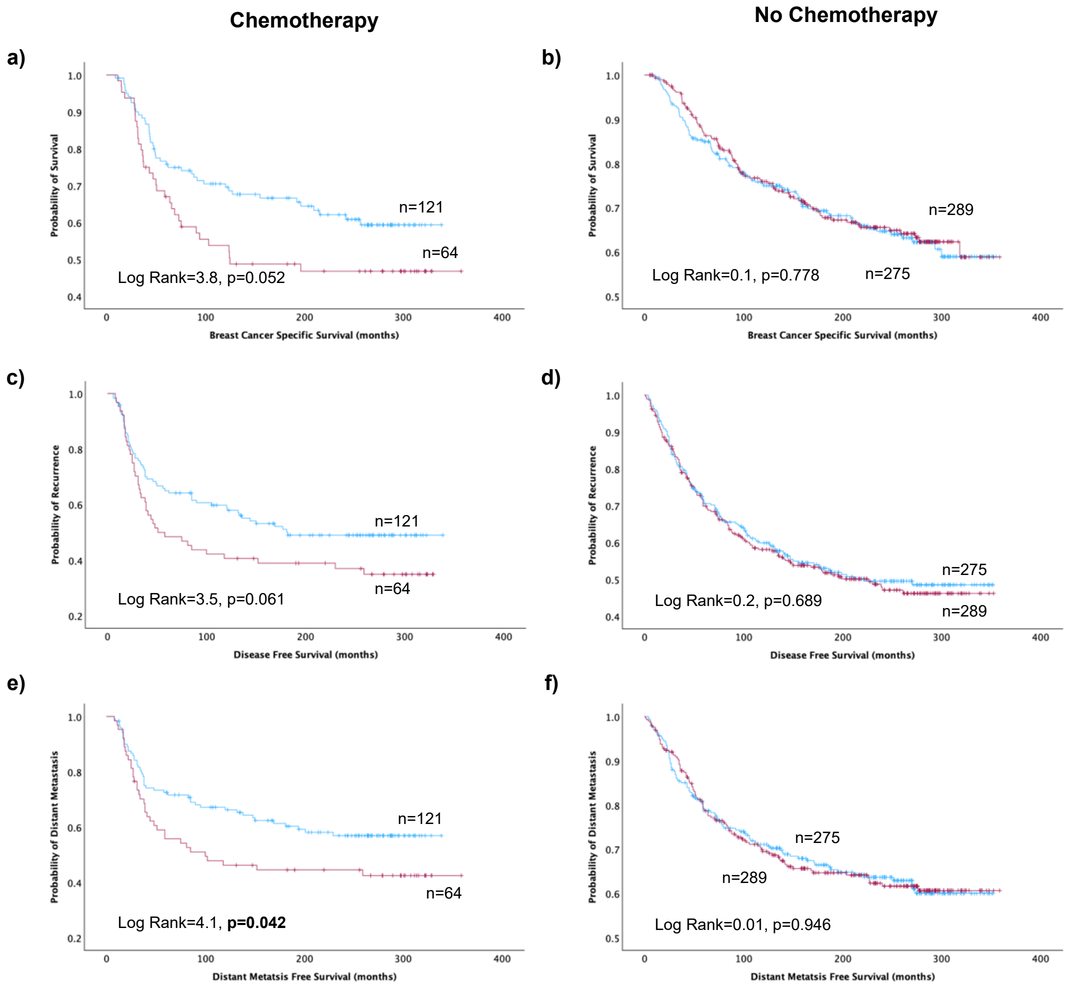

Supplementary Figure 6. Kaplan-Meier estimates of TRIM2 protein expression in breast cancer treated with or without chemotherapy: a-b) Breast Cancer Specific Survival, c-d) Disease Free Survival, e-f) Distant Metastasis Free Survival. TRIM2 high (red line) and low (blue line)

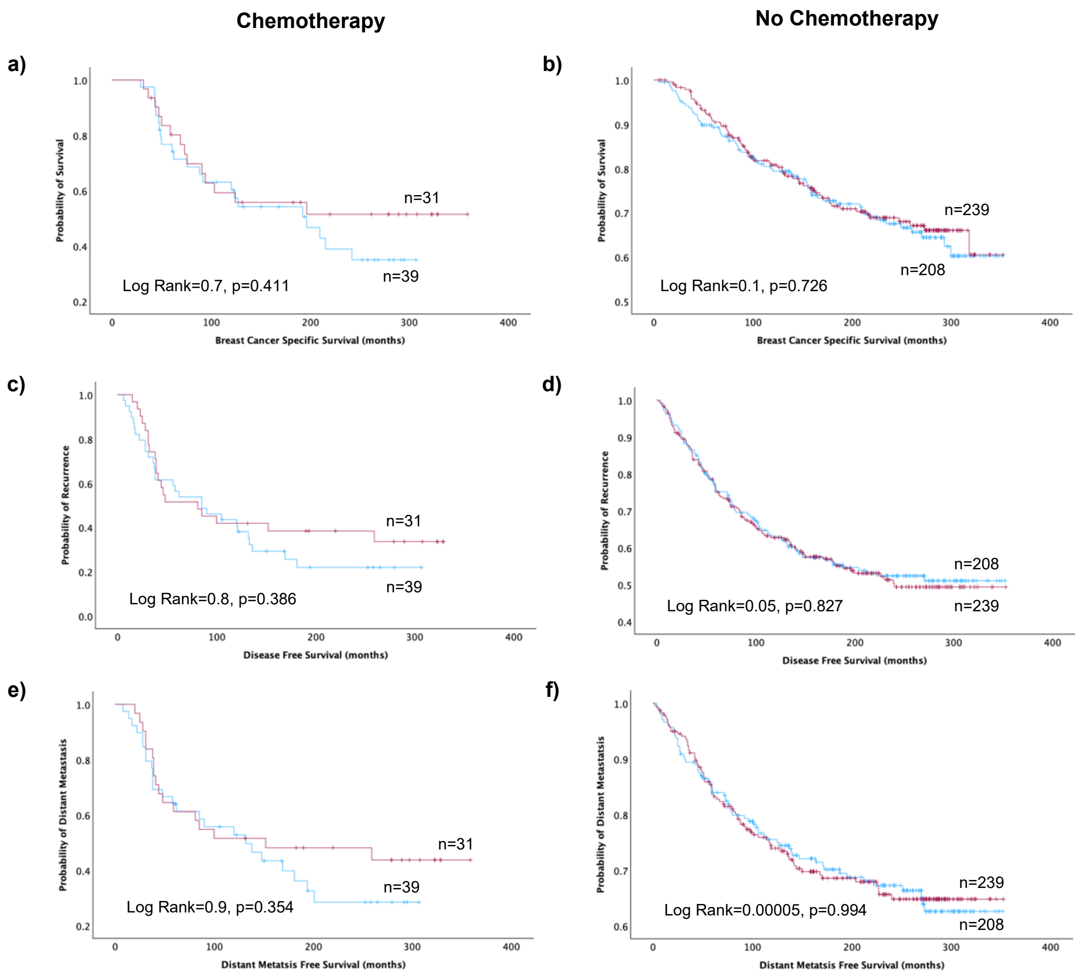

Supplementary Figure 7. Kaplan-Meier estimates of TRIM2 protein expression in Estrogen Receptor positive breast cancer treated with or without chemotherapy: a-b) Breast Cancer Specific Survival, c-d) Disease Free Survival, e-f) Distant Metastasis Free Survival. TRIM2 high (red line) and low (blue line)

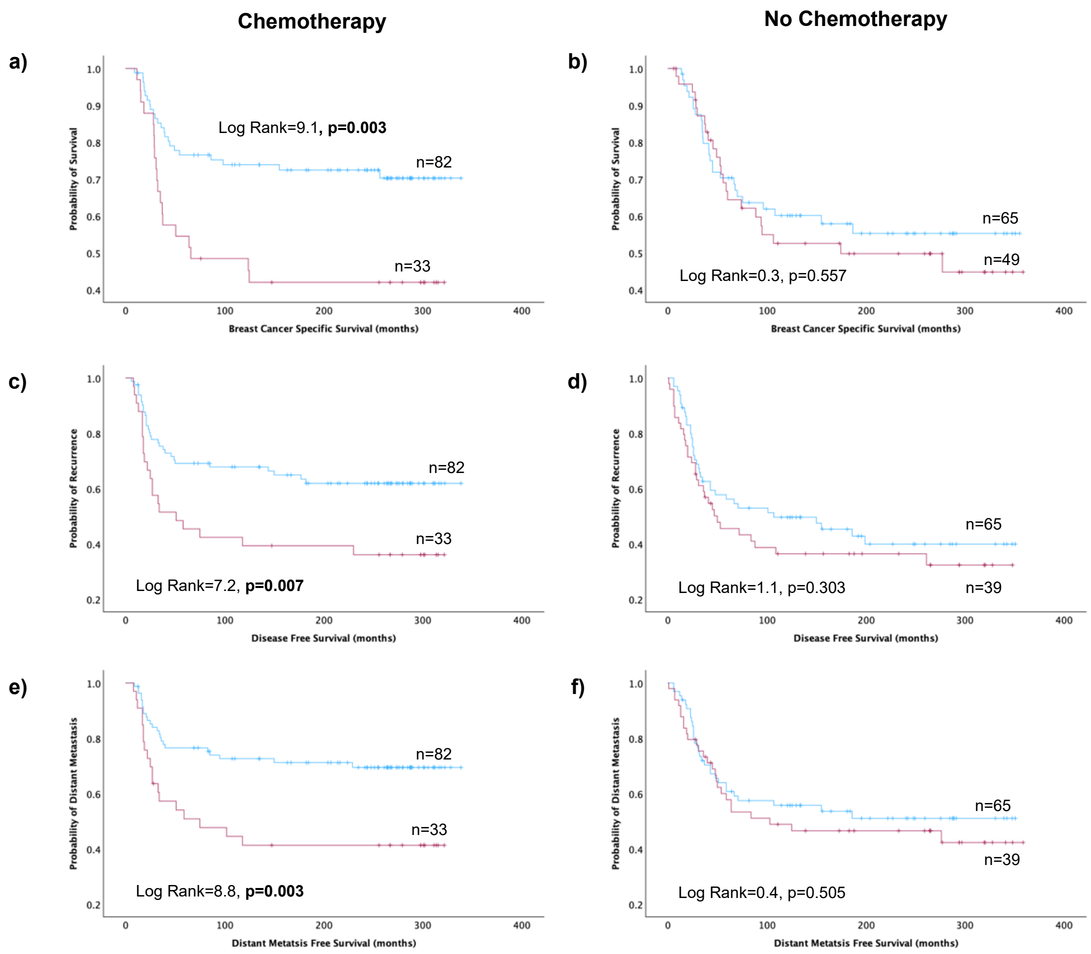

Supplementary Figure 8. Kaplan-Meier estimates of TRIM2 protein expression in Estrogen Receptor negative breast cancer treated with or without chemotherapy: a-b) Breast Cancer Specific Survival, c-d) Disease Free Survival, e-f) Distant Metastasis Free Survival. TRIM2 high (red line) and low (blue line)

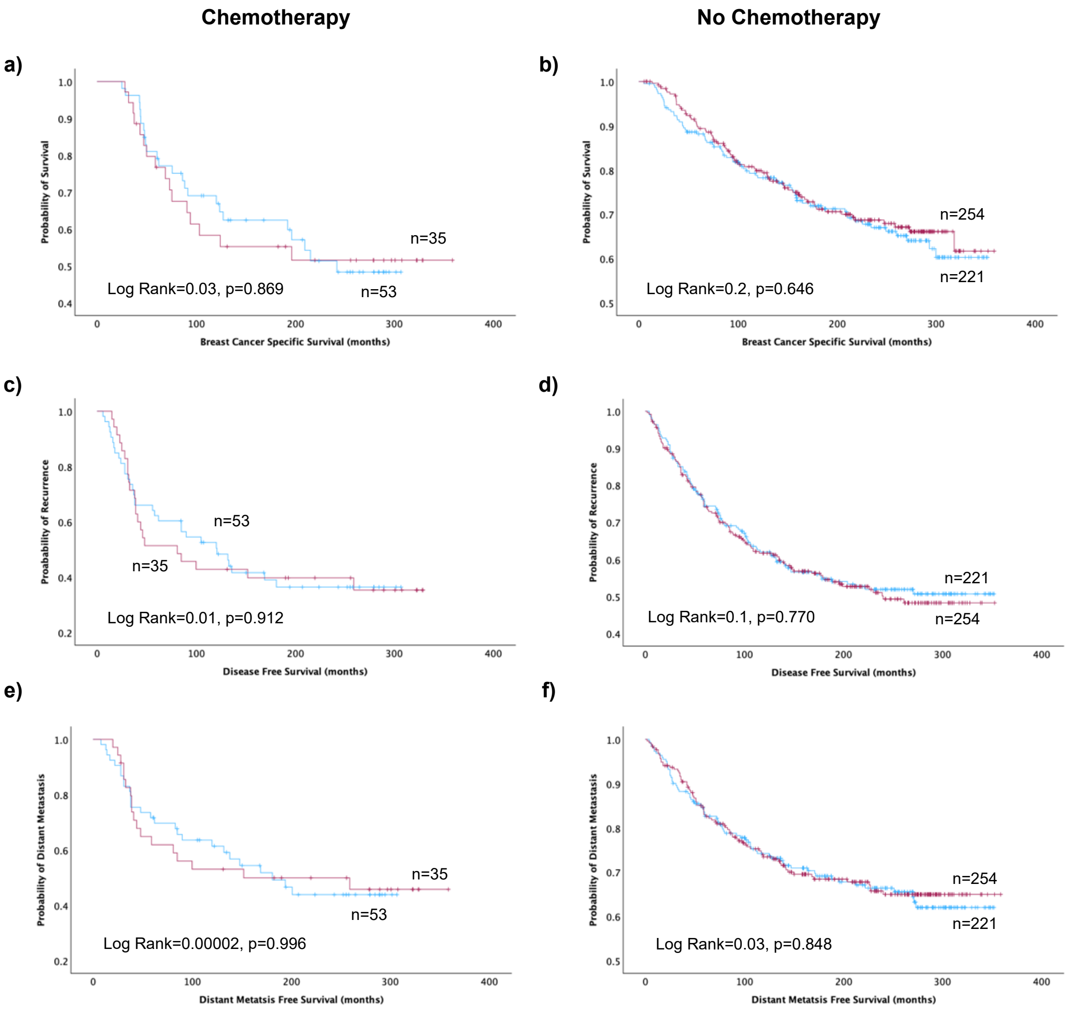

Supplementary Figure 9. Kaplan-Meier estimates of TRIM2 protein expression in non-triple negative breast cancer treated with or without chemotherapy: a-b) Breast Cancer Specific Survival, c-d) Disease Free Survival, e-f) Distant Metastasis Free Survival. TRIM2 high (red line) and low (blue line)
